# PReS-FINAL-2021: JADAS-CRP instead of JADAS-ESR...results from REUMA.PT

**DOI:** 10.1186/1546-0096-11-S2-P34

**Published:** 2013-12-05

**Authors:** AF Mourão, MJ Santos, J Melo Gomes, F Martins, JA Costa, F Ramos, I Brito, C Duarte, R Figueira, G Figueiredo, C Furtado, A Lopes, M Oliveira, A Rodrigues, M Salgado, M Sousa, J Branco, J Eurico Fonseca, H Canhão

**Affiliations:** 1 CEDOC - Centre for Chronic Diseases, Faculdade de Ciencias Medicas da Universidade Nova de Lisboa, Portugal; 2 Rheumatology Research Unit, Instituto de Medicina Molecular, Lisbon, Portugal; 3 Rheumatology, Hospital Egas Moniz, Lisbon, Portugal; 4 Rheumatology, Hospital Garcia de Orta, Almada, Portugal; 5 Pediatric Rheumatology, Instituto Portugues de Reumatologia, Portugal; 6 Rheumatology, Sociedade Portuguesa de Reumatologia, Lisbon, Portugal; 7 Rheumatology, Hospital Conde de Bertiandos - ULSAM, Ponte de Lima, Portugal; 8 Rheumatology, Hospital de Santa Maria, Lisbon, Portugal; 9 Rheumatology, Hospital de São João, Porto, Portugal; 10 Rheumatology, Centro Hospitalar de Coimbra, Coimbra, Portugal; 11 Rheumatology, Hospital Nélio Mendonça, Funchal, Portugal; 12 Rheumatology, Hospital do Divino Espírito Santo, São Miguel, Portugal; 13 Rheumatology, Centro Hospitalar da Cova da Beira, Covilhã, Portugal; 14 Rheumatology, Hospital de Angra do Heroísmo, Terceira, Portugal; 15 Pediatrics, Centro Hospitalar de Coimbra, Hospital Pediátrico, Coimbra, Portugal; 16 Rheumatology, Instituto Portugues de Reumatologia, Lisbon, Portugal

## Introduction

Recently, Juvenile Arthritis Disease Activity Score (JADAS) was found to be a valid instrument for assessment of disease activity. JADAS was developed with erythrocyte sedimentation rate (ESR) because C-reactive protein (CRP) values were not available in all databases used to validate the tool. *Nordal et al *compared recently in a Nordic population the JADAS based on CRP with JADAS based on ESR and concluded that these instruments correlated closely, indicating that both scores can be recommended for assessing disease activity in JIA.

## Objectives

Determine JADAS-CRP and compare its performance to the JADAS-ESR and to test the agreement of both scores on each disease activity category, in a Portuguese population with JIA.

## Methods

A National cohort of patients with JIA, registered in Rheumatic Diseases Portuguese Register, was selected. Patients were included in the study when all disease activity measures were available for JADAS-ESR and CRP calculation and one visit per patient was randomly selected. JADAS-CRP was adapted by replacing ESR with CRP as the inflammatory marker. CRP was truncated to a 0-10 scale, similar to the truncated ESR used in JADAS. JADAS 27-CRP was calculated similarly to JADAS 27-ESR as the simple linear sum of its four components. Pearson correlations and K statistics were used in analyses.

## Results

358 children included, 65.4% were female, mean disease duration 11.75 ± 9.03 years. 37,5% were persistent oligoarticular, 14,8% had extended oligoarticular, 14,2% were polyarticular rheumatoid factor negative, 8,4% polyarticular RF positive, 10,9% systemic, 9,8% enthesitis-related arthritis, 3,1% psoriatic arthritis and in 1,4% patients we could not have access to the subtype of JIA. The correlation coefficient was 0,967, *p *< 0,0001), though the correlation coefficient between CRP and ESR was only 0,335 (p < 0,0001). When comparing the JADAS-ESR and JADAS-CRP within each subtype of JIA, the strong correlation was maintained (all values of correlation > 0.9 and all p-values < 0.0001). The agreement between JADAS-ESR and CRP across the 4 activity states (inactive disease, minimal disease activity, parent's acceptable symptom state and active disease) assessed by K statistics was very good, showing 91.1% of the observations in agreement, K = 0.867 (95%CI 0.824-0.91).

## Conclusion

In our study the JADAS27 based on CRP and ESR correlated closely, indicating that both measures can be used in clinical practice.

## Disclosure of interest

None declared.

